# Alleviating premenstrual symptoms with smartphone-based heart rate variability biofeedback training: a pilot study

**DOI:** 10.3389/fdgth.2024.1337667

**Published:** 2024-06-14

**Authors:** Berenike Lisa Blaser, Mathias Weymar, Julia Wendt

**Affiliations:** ^1^ Department of Biological Psychology and Affective Science, Faculty of Human Sciences, University of Potsdam, Potsdam, Germany; ^2^ Faculty of Health Sciences Brandenburg, University of Potsdam, Potsdam, Germany

**Keywords:** smartphone photoplethysmography, mHealth, heart rate variability, biofeedback, premenstrual syndrome, stress, depression, attentional control

## Abstract

**Introduction:**

Heart rate variability biofeedback (HRVB) is a well-studied intervention known for its positive effects on emotional, cognitive, and physiological well-being, including relief from depressive symptoms. However, its practical use is hampered by high costs and a lack of trained professionals. Smartphone-based HRVB, which eliminates the need for external devices, offers a promising alternative, albeit with limited research. Additionally, premenstrual symptoms are highly prevalent among menstruating individuals, and there is a need for low-cost, accessible interventions with minimal side effects. With this pilot study, we aim to test, for the first time, the influence of smartphone-based HRVB on depressive and premenstrual symptoms, as well as anxiety/stress symptoms and attentional control.

**Methods:**

Twenty-seven participants with above-average premenstrual or depressive symptoms underwent a 4-week photoplethysmography smartphone-based HRVB intervention using a waitlist-control design. Laboratory sessions were conducted before and after the intervention, spaced exactly 4 weeks apart. Assessments included resting vagally mediated heart rate variability (vmHRV), attentional control via the revised attention network test (ANT-R), depressive symptoms assessed with the BDI-II questionnaire, and stress/anxiety symptoms measured using the DASS questionnaire. Premenstrual symptomatology was recorded through the PAF questionnaire if applicable. Data analysis employed linear mixed models.

**Results:**

We observed improvements in premenstrual, depressive, and anxiety/stress symptoms, as well as the Executive Functioning Score of the ANT-R during the intervention period but not during the waitlist phase. However, we did not find significant changes in vmHRV or the Orienting Score of the ANT-R.

**Discussion:**

These findings are promising, both in terms of the effectiveness of smartphone-based HRVB and its potential to alleviate premenstrual symptoms. Nevertheless, to provide a solid recommendation regarding the use of HRVB for improving premenstrual symptoms, further research with a larger sample size is needed to replicate these effects.

## 1 Introduction

Heart rate variability biofeedback (HRVB) is a well-researched intervention that has demonstrated effectiveness in a wide range of areas (1), including relieving anxiety and stress (2), ameliorating depression (3), improving sleep (4), alleviating asthma symptoms (5), and even enhancing sports performance (6). However, despite its potential, this user-friendly method has seen limited practical implementation. This can be attributed, in part, to the high costs associated with necessary stationary and mobile electrocardiography (ECG) devices, as well as the required training and expertise of staff members entrusted with its administration, which further strains healthcare systems. Encouragingly, smartphone apps capable of assessing heart rate through the device's camera, without additional equipment, are promising to yield similar results (7). Nevertheless, empirical validation of smartphone-based HRVB applications remains limited (see below for elaboration). This study aims to validate the effectiveness of an HRVB intervention applied through smartphones, specifically targeting the alleviation of depressive symptoms, a well-documented outcome of conventional HRVB. Additionally, we explore a novel application of HRVB for premenstrual symptoms.

HRVB is a method in which vagally mediated heart rate variability (vmHRV), an indicator of parasympathetic activity (8, 9), is systematically increased through slow, controlled breathing and visual feedback of heart rate oscillations. The primary driving mechanism involves slow-paced breathing at 0.1 Hz or an individual resonance frequency (10). It is believed to exert its various beneficial effects through bottom-up modulation of a neural network described by Thayer and Lane (11) in their neurovisceral integration model.

This model delineates a network of interconnected structures known as the central autonomic network (CAN), responsible for integrating information and regulating appropriate responses. At the core of this regulatory network, Thayer and Lane (12) propose an inhibitory connectivity between the medial prefrontal cortex (mPFC) and the amygdala. The stronger this connectivity, the greater an individual's capacity to downregulate a presumed default stress response and deliver a precise and personalized reaction to internal and environmental demands. vmHRV is considered both a peripheral index for this capacity and a reciprocal element within this network (13). This theory is grounded in a substantial body of evidence linking low vmHRV to psychopathology (14) and reduced performance in cognitive self-control tasks (15, 16).

When practiced over several weeks, HRVB enhances the capacity of the CAN through coherence phenomena involving the synchronization of breathing rate, blood pressure, and heart rate oscillations (17). These phenomena contribute to several bottom-up routes. The most crucial of these routes involve input into the CAN through baroreceptors via the nucleus of the solitary tract, stretch receptors in the lungs, and a vagal afferent pathway (17–19).

HRVB interventions have demonstrated the potential to improve various affective and cognitive outcomes associated with CAN capacity, including depression (3), anxiety (2), mind wandering (20) and inhibitory control (21). Our study aims to expand these effects in the context of a smartphone-based intervention. While vmHRV is reliably associated with cognitive outcomes, particularly executive functions, the impact of HRVB on these variables is less clear (22). In a systematic review, Tinello et al. (22) found that existing effects are primarily observed in the domain of attentional control and are often found in patient populations or individuals experiencing high levels of stress. Given that attention is strongly linked to vmHRV, we also investigated the effect of HRVB on attentional control using the revised Attention Network Test [ANT-R, (23, 24)].

Expanding on these replications, we further investigate HRVB impact on premenstrual syndrome (PMS), a highly prevalent condition characterized by a diverse collection of psychological and physiological symptoms. These symptoms typically manifest in individuals with active menstrual cycles during the week leading up to menstruation and tend to subside shortly after. As many as 90% of menstruating individuals regularly encounter at least one symptom of PMS (25). Commonly reported symptoms encompass heightened stress reactivity, anxiety, depressive mood, breast tenderness, and abdominal pain (26, 27).

As a component of the gender data gap, premenstrual syndrome (PMS) remains significantly under-researched (28). Even today, treatment options remain limited, primarily centered on addressing specific psychological or physiological symptoms through hormonal cycle suppression or antidepressant medication in both clinical practice and research (29). Both of these approaches are associated with substantial adverse side effects (30–32).

Premenstrual symptoms have been linked to cyclic fluctuations in vmHRV (33). Individuals who experience more severe symptoms tend to exhibit a pronounced reduction in vmHRV during the luteal phase of their menstrual cycle, coinciding with the experience of these symptoms (34). Matsumoto et al. (34) have suggested a potential causal relationship in this regard.

One possible explanation for this phenomenon lies in a metabolite of progesterone, one of the main fluctuating gonadal steroids during the menstrual cycle. Sundström-Poromaa et al. (35) have identified this metabolite, namely Allopregnanolone (ALLO), an allosteric Gamma-Aminobutyric Acid (GABA) receptor modulator as a likely cause of the experience of premenstrual symptoms (27). As ALLO operates on the GABAergic system, the proposed CAN in the neurovisceral integration theory (11, 12) might also be affected. In this theory, successful adaptation relies on inhibitory connectivity between the mPFC and the amygdala. The strength of these connections, which are part of the central nervous system's inhibitory GABAergic network, are influenced by GABA levels in the mPFC (36). Compromised inhibition in this circuit due to ALLO withdrawal and/or maladaptive ALLO responses may lead to a compromised self-regulatory capacity of the organism on both affective and physiological levels, as observed in PMS.

Following this line of reasoning, HRVB is a promising candidate to counteract some of these effects through two mechanisms. Firstly, the most pronounced effects of HRVB are observed in stress management (2). If stress throughout the cycle causes irregularities in the ALLO system during the premenstrual phase, reducing stress throughout the cycle may prevent some of the symptom development. Existing evidence already suggests that various relaxation techniques can positively impact PMS (37). Secondly, HRVB is assumed to increase the inhibitory capacity of the mPFC over the amygdala and, as a result, enhance the inhibition of the default stress response (38). Although GABAergic transmission may be compromised during the premenstrual phase, boosting the baseline inhibitory strength between these two brain structures could raise inhibition levels. This might make it less likely for a sudden drop to cross the threshold to trigger symptoms that cause significant distress.

Initial studies have already provided evidence of the effectiveness of HRVB for mental health outcomes when administered through smartphones. Previous studies that utilized smartphone-based HRVB interventions to improve outcomes like depressive or anxiety symptoms, however, have typically relied on external devices connected to the smartphone via Bluetooth. These devices include wearable ECG-measuring chest straps (38–42) or earlobe-clip pulse measuring devices (43–45). Acquiring a wearable device presents a significant obstacle for potential HRVB users.

Smartphone cameras can now measure heart rate when the user places a finger on the camera. An application activates the camera flash and analyzes the red-to-green ratio in the image at high frequency, generating pulse curves. This process is known as photoplethysmography (PPG) and closely resembles the process behind the optical sensors that emit infrared or green light in commonly used pulse measurement devices. Yuda et al. (7) suggest that the heart rate variability indicator used in smartphone apps, which they term “pulse rate variability” as measured through PPG, may contain distinct information compared to its ECG-measured counterpart. Nevertheless, recent research has demonstrated very high Pearson correlations between HRV parameters measured through ECG and PPG of *r* > .9 (46), even though the reliability is somewhat dependent on sampling rate of the device (47). Moreover, the associations with mental health outcomes are also evident when assessing vmHRV via PPG using the smartphone camera (48). This supports the use of PPG as a foundation for HRVB.

In this study, we investigated the novel application of a 4-week smartphone-based HRVB intervention using PPG via smartphone camera instead of an external device for alleviating depressive and premenstrual symptoms. Our sample comprised young adults who either exhibited above-average PMS or depressive symptoms. Additionally, we examined the impact of the intervention on various other outcomes, including anxiety and stress symptoms, attentional control, and vmHRV. Our hypotheses were as follows: After a 4-week smartphone-based HRVB intervention using PPG, premenstrual symptoms (H1), depressive symptoms (H2), and anxiety/stress symptoms (H3) will be reduced, while no changes will be observed during a 4-week waitlist period. Additionally, vmHRV (H4) and attentional control (Orienting attention component H5a and Executive Functioning attention component H5b) will improve during a 4-week smartphone-based HRVB intervention using PPG, while no changes will be observed during a 4-week waitlist period.

## 2 Methods

### 2.1 Participants

A G*Power analysis revealed that a sample size of 40 was necessary to detect an effect size of.4, based on a meta-analytic effect of HRVBFB on depressive symptoms reported by Pizzoli et al. (3), with a power of .8 and a one-tailed alpha error probability of .05. However, due to recruitment difficulties and resourcing issues by the company providing the app during the extended recruitment period, we were unable to reach our target of 40 participants.

Twenty-nine participants were recruited from the student population of the University of Potsdam for this study. Recruitment was carried out via the online recruiting platform for study participants of the cognitive sciences (Sona Systems, https://www.sona-systems.com) of University Potsdam as well as via flyers on campus and advertisement in university mailing lists. Inclusion criteria required participants to have either above-average premenstrual symptomatology (short version Premenstrual Assessment Form, PAF20 ≥ 50), depressive symptoms that indicate at least minimal depression (Beck's Depression Inventory, BDI-II ≥ 9), or both. Participants who exceeded a BDI-II score of 14 received a consultation with a clinical psychologist to discuss possible necessary treatment prior to study participation.

Exclusion criteria included factors proposed by Laborde et al. (49) such as pregnancy, heart rate-altering chronic diseases or medication. We additionally excluded competitive athletes to avoid ceiling effects, since this population has systematically increased vmHRV (50). Participants currently in any treatment or planning significant lifestyle changes during the period of study participation were also excluded. In addition, participants were required to be at least 18 years of age.

All participants provided informed consent prior to their inclusion for a study protocol approved by the ethics committee of the University of Potsdam (No. 30/2022). Participants who met the inclusion criteria were eligible for study participation and received either course credits or monetary compensation.

### 2.2 Procedure

The study protocol was preregistered on Open Science Framework (osf.io/68fzq). The study procedure began with an online screening questionnaire to determine eligibility based on inclusion and exclusion criteria, as well as to assess sociodemographic factors such as age, gender, study program, and BMI. Participants were also required to provide information about their menstrual cycle to ensure that the appropriate questionnaires were administered. Additionally, participants were asked to provide their email address for communication throughout the study.

All eligible participants took part in a 4-week biofeedback intervention during which they practiced smartphone-based HRVB for at least 5 min every day. After the first and second week, participants additionally received an online coaching session to improve their technique and address any technical or other difficulty they encountered.

Before and after the 4-week intervention, participants completed laboratory sessions that were scheduled at the same time, exactly 4 weeks apart (T1 and T5). During these sessions, various measures were collected, including vagally mediated heart rate variability by ECG, attentional control using the reaction time paradigm ANT-R (24), and self-reported symptoms of depression, premenstrual syndrome (PMS), and anxiety/stress via questionnaires.

To ensure balanced allocation of participants to the waitlist group, half of the participants within each group of inclusion criteria (depression, PMS, or both) were pseudo-randomly assigned to the waitlist group. For each inclusion criterion, blocks of 8 group assignments (waitlist/intervention), counterbalanced with the block order of the ANT-R, attached to a participant code, were shuffled and then consecutively assigned to the recruited participants. The creation of the allocation sequence was conducted by the first author. The recruitment and assignment of participant codes, and thus group assignment, were carried out by the staff members conducting the investigation. The waitlist group additionally completed a laboratory session four weeks prior to study inclusion, during which the same parameters were assessed (W1).

**Figure 1:**
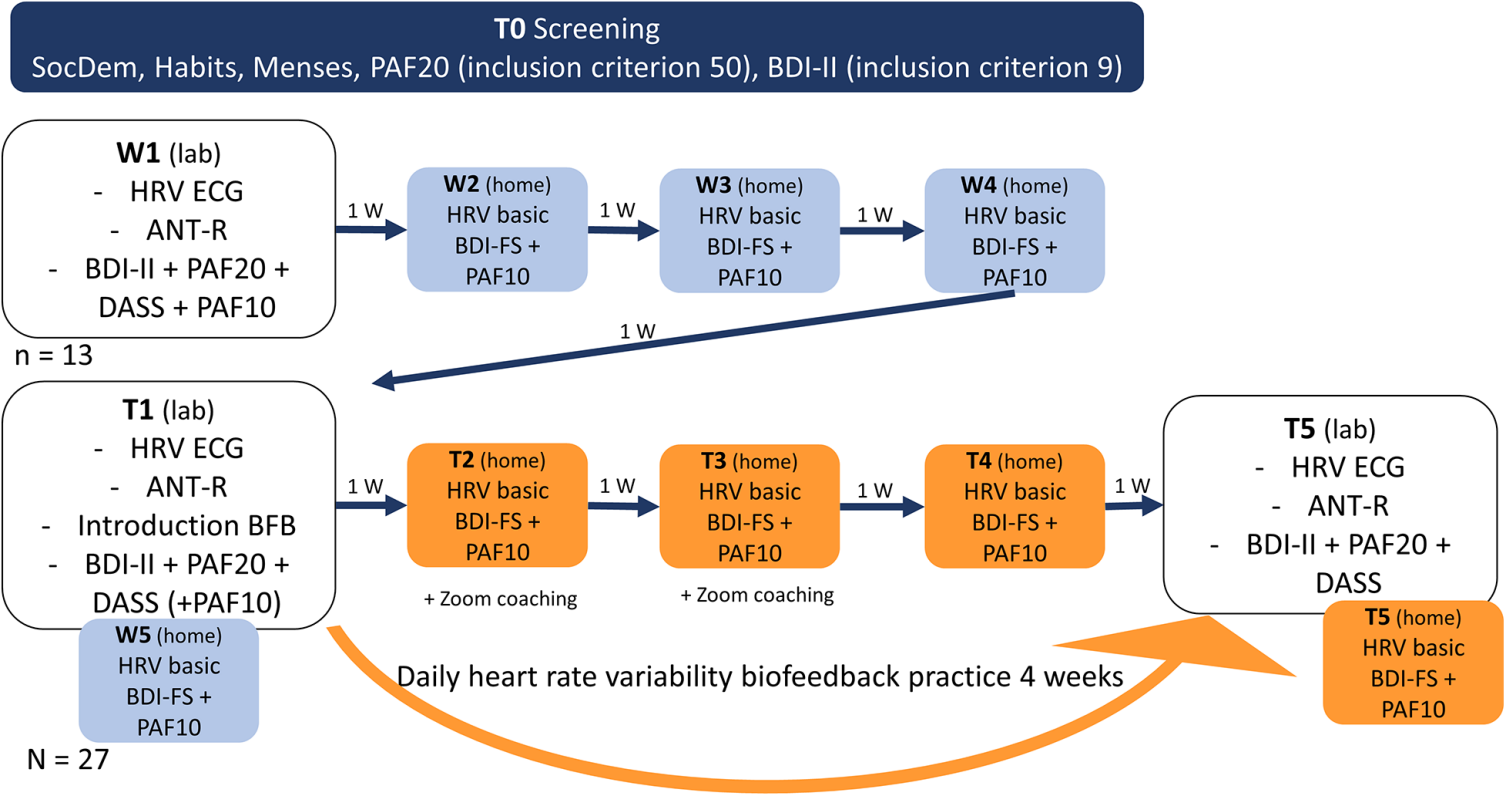
Study procedure. SocDem, sociodemographic information; PAF10, premenstrual assessment form 10 item version; PAF20, premenstrual assessment form 20 item version; BDI-II, Beck's depression inventory; BDI-FS, Beck's depression inventory short version; ANT-R, attention network test-revised; DASS, Depression Anxiety Stress Scale; HRV ECG, heart rate variability measurement in lab (electrocardiography); HRV basic, measurement at rest at home on phone (photoplethysmography); W, week.

Throughout the study, participants completed short versions of the depressive and premenstrual symptom questionnaires and underwent a photoplethysmography based HRV measurement at home using the biofeedback app, each week on the same day and at the same time that they chose (W2-W4 and T2-T4). Participants received automated email reminders and a link to the respective questionnaire to ensure compliance. The results of these measurements are not analysed and reported in this report in order to maintain clarity and comprehensiveness in the manuscript. Figure 1 provides an overview of the study procedure.

All participants received an introduction to smartphone-based HRVB (app provided by Kenkou GmbH) followed by a training period during T1. The waitlist group received a tutorial on conducting vmHRV measurements at home with the app during W1, while the intervention-only group received this tutorial during T1. Participants were blinded to the intervention allocation during the first session until all measurements were taken.

Since PMS occurs only once during each menstrual cycle, and cycle lengths can vary significantly both between and within individuals, we included a follow-up measurement of the online questionnaire 4 weeks after T5. If a participant reported no new menstruation onset during the last two weeks of the intervention, indicating no new premenstrual phase, we used the PMS values reported in the follow-up measurement as the post-intervention values, describing the next premenstrual phase after completing the intervention.

### 2.3 Smartphone-based heart rate variability biofeedback

The HRVB intervention used in this study was app-based and built with the software development kit (SDK) provided by Kenkou GmbH. The app measured HRV via photoplethysmography (PPG), whereby participants placed their index finger on the camera lens and a flash was used to illuminate the tissue. The camera measured the intensity of blood flow, and a peak detection algorithm was used to detect heartbeats.

**Figure 2:**
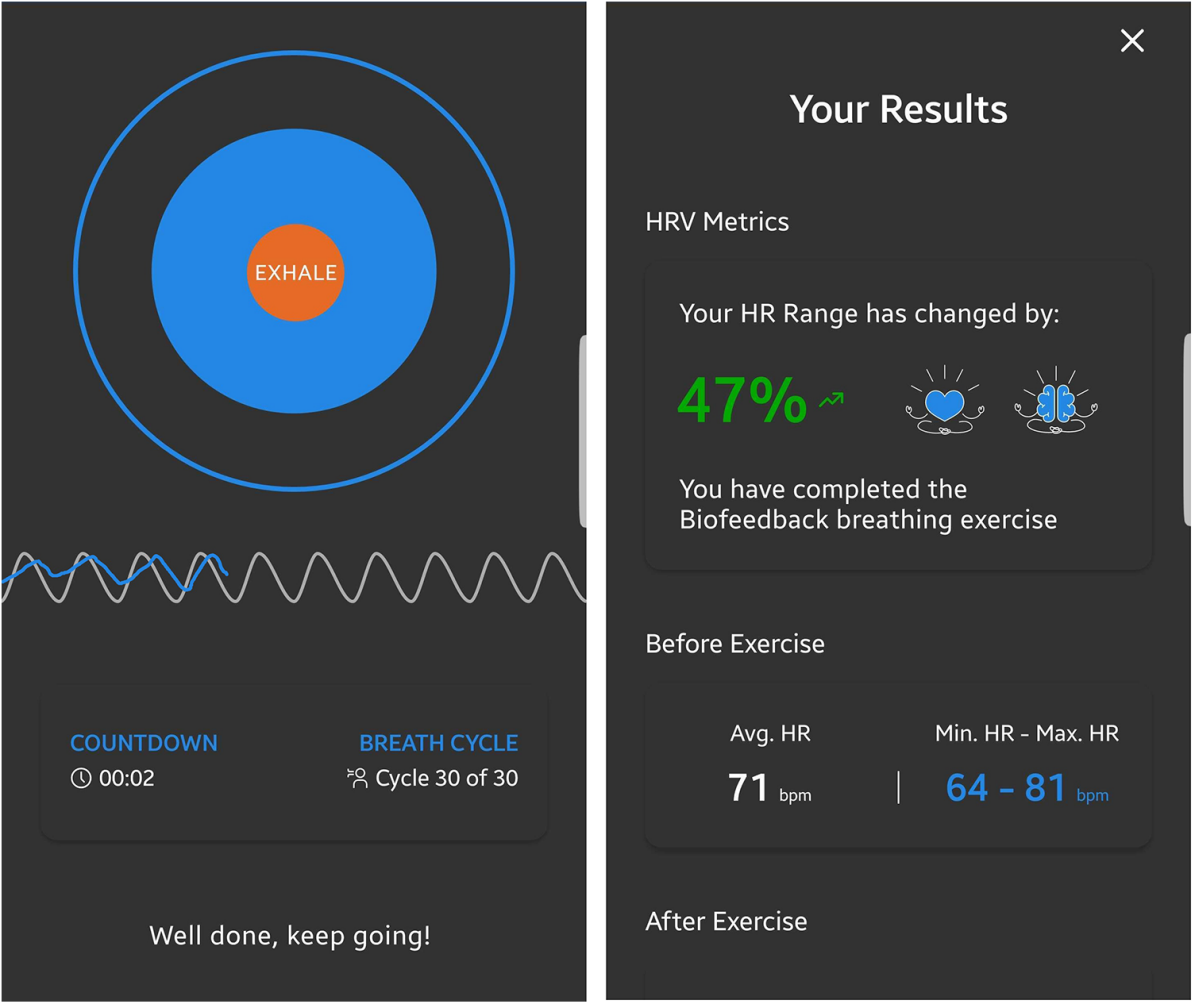
Screenshots of the HRVB application. For the biofeedback, the current respiratory sinus arrhythmia, measured through photoplethysmography using the device's integrated camera, was represented as a blue line. A dynamic expanding and contracting circle visually represented the paced breathing rhythm at a frequency of 0.1 Hz. This frequency was further depicted by gray sinusoidal waves in the background, behind the measured heart rate oscillations.

Before each biofeedback session, participants underwent a 1-min baseline measurement to assess their current state, which allowed the feedback to be adjusted accordingly. During all measurements, automated quality assessment provided feedback on the quality of the PPG signal. If measurement quality was too low, the recording was paused and the participant received instructions on how to improve the signal in the app. During the biofeedback session, participants saw a growing and shrinking circle indicating the breathing rhythm at 0.1 Hz. A fixed sinus-like wave was also displayed at the frequency of the paced breathing, and the pulse rate over time was mapped on top of this. Participants were instructed that the two waves would converge more as they relaxed. After the HRVB exercise, the user was given feedback on HRV improvement during the exercise to increase motivation. Screenshots of the app can be seen in Figure 2.

The experimenter provided approximately 15 min of instructions to participants on how to use the app and how the biofeedback worked. Participants were coached on how to engage in relaxed slow-paced breathing. They were instructed to practice at least 5 min daily for the next 4 weeks, with the option to practice for longer periods of time if desired. Participants were also informed that more practice would likely lead to greater benefits.

Three participants encountered technical difficulties while running the application on their devices. To ensure their participation in the intervention, they were provided with an alternative mobile HRVB system (“Qiu” by Biosign®, D-85570, Ottenhofen, Germany).

### 2.4 Outcome measures

#### 2.4.1 Premenstrual assessment form (short form)

The short form of the Premenstrual Assessment Form (PAF20) is a retrospective instrument that assesses PMS symptoms during the last premenstrual phase (26). It was derived from the 20 most endorsed items of the long form PAF, which includes almost 100 items (51). Each item represents one premenstrual symptom, for which the participant must indicate how strongly they experienced it during the last cycle on a 6-point Likert scale from 1 (not at all/no change) to 6 (extreme change). The German translation of the PAF-20 shows good internal consistency and reliability and loads on two factors, indicating a psychological and physiological subscale (52).

The 10-item version (PAF-10) was constructed using the items with the highest factor loadings and shows a very high correlation with the PAF-20 (52). To assess the fluctuations of symptoms throughout the cycle and approximate a prospective assessment, the participants filled out the PAF-10 once a week with altered instructions, asking for a report of the 10 symptoms during the last week.

#### 2.4.2 Becks depression inventory

The Beck Depression Inventory II (BDI-II) is a widely used questionnaire that assesses the severity of depressive symptoms. It consists of 21 items, each containing four statements about depressive symptoms ranging from 0 (normal) to 3 (most severe). The total maximum score is 63. The BDI-II has good psychometric properties, including high internal consistency, test-retest reliability, and concurrent and discriminant validity. Additionally, the questionnaire has been translated into multiple languages and is widely used in clinical and research settings to assess depression severity, monitor treatment progress, and evaluate outcomes. Previous studies have also shown that the BDI-II has good discrimination between patients with varying degrees of depression and accurately reflects changes in depression intensity over time (53, 54).

The Fast Screen Version of the Becks Depression Inventory (BDI-FS) was developed as a short form to allow for parsimonious screenings, e.g., in research settings. It includes seven items and is based on the DSM-5 criteria for depression, clinical importance, and factor loadings (55).

#### 2.4.3 Depression Anxiety Stress Scale

The German version of the Depression Anxiety and Stress Scale (DASS), developed by Henry and Crawford (56) and based on the original version by Lovibond and Lovibond (57), was employed for data collection. The DASS-21, a shortened version of the scale, consists of 21 statements that assess three distinct subscales: depression, anxiety, and stress.

Participants were asked to rate the extent to which each statement applied to them during the designated period using Likert scales ranging from 0 to 3. Higher scores on the DASS-21 indicate elevated levels of depressive symptoms, anxiety, and stress.

The internal consistency of the DASS-21 was found to be satisfactory, with a Cronbach's *α* coefficient of 0.89 (58). The DASS-21 was selected as an outcome measure in this study based on its consistent effects in biofeedback interventions, as demonstrated in prior research (2).

#### 2.4.4 Vagally mediated heart rate variability

Resting vmHRV was determined using the BioSign software and hardware (“HRV-Scanner”; Biosign®, D-85570, Ottenhofen, Germany). Participants had been sitting down for at least 15 min before the measurement. The measurement was taken in a sitting position. Participants were instructed to sit comfortably, place their feet side by side on the floor, close their eyes, and were told that they didn't have to pay attention to anything in particular. Following the recommendations by Laborde et al. (49), the measurement had a duration of 5 min.

HRV was measured by a one-lead electrocardiogram (ECG) through two surface sensors attached to the right and left wrists of the participant. The device worked with a sampling rate of 500 Hz and a 16-bit resolution. Artifacts and abnormal beats were filtered in a two-step process following the software documentation (59). First, the HRV Scanner software automatically marked areas of the heart rate curve that included implausible changes in heart rate (through the division of the heart rate curve into small segments and a subsequent scan of each segment). This process was based on an algorithm patented by the BioSign company that identifies outliers in a Poincaré plot, where each RR interval is plotted against the previous RR interval.

Working with these recognized areas of possible disturbances, in the second step, the R-spike recognition was manually assessed and corrected, and artifacts (e.g., due to movement) were removed. After the two-step process, the data quality was excellent, with less than 0.1% artifacts per measurement on average.

Participants additionally conducted short resting vmHRV measurements through the app using PPG, as described above, once a week. These measurements lasted one minute, and participants were instructed to take these measurements each week on the same day, at the same time, and in the same place, ensuring they would not be disturbed. They were also instructed to sit comfortably and close their eyes during the measurement, similar to the way they were during the ECG measurement in the laboratory.

We used the root mean square of successive differences (RMSSD) as a measure for vagally mediated heart rate variability. This choice was due to its indication of parasympathetic output and robustness to influences of breathing rate (60).

#### 2.4.5 Attentional network test revised

We employed the ANT-R, developed by Fan et al. (24), as a measure of attentional control. This task is reaction time task and was designed as a combination of the Eriksen flanker task (61) and the Posner cueing task (62).

During the ANT-R, participants were presented with a grey background and a black horizontal arrow. Their task was to indicate the direction of the arrow by pressing the corresponding button with their left or right index finger.

The ANT-R task consists of a total of 288 trials, divided into two identical runs of 144 trials each. The duration of the entire test is approximately 30 min. Previous studies have demonstrated good split-half reliability in the Executive (*r* = .74) and Orienting network scores (*r* = .70) (63). To reduce participant burden, only one run was completed per session.

The task was administered using the Presentation® software (Neurobehavioral Systems, Inc.) on a 24-inch screen positioned 80 cm away from the participants. Before the main task, participants completed 6 practice trials with feedback and 32 practice trials without feedback. Written and visual instructions were provided prior to the practice trials.

During the main task, participants were required to achieve a minimum accuracy of 80%. On average, participants reached an accuracy rate of 95% in the main task block.

### 2.5 Statistical analysis

All analyses were conducted using R (version 4.2.2). A linear mixed model was calculated for each target variable, with data points clustered per participant by introducing participant intercepts as random effects. When applicable, items were also included as random effects. The fixed effects included in the model were TIME point, TREATMENT, the TREATMENT * TIME interaction, and control variables (AGE, GENDER, BMI, RMSSD), along with exploratory three-way interactions involving potential mediators of the main TREATMENT * TIME effect.

A model selection process was applied to each analysis, with predictors being consecutively added to the model. Likelihood Ratio Testing compared the goodness of fit of each model to the next simpler one, and predictors were retained if they improved the model's fit.

The main hypotheses were tested with TREATMENT * TIME interactions in the respective model. The hypothesis was considered accepted if the interaction term was included in the final model, a significant predictor, and the effect aligned with the expected direction. *Post hoc* comparisons and plots of the interaction effects were used to verify the expected direction of the effects.

As this study was a randomized controlled trial (RCT) with a waiting-list control group, the analyses included post-treatment data from the control group. Therefore, the post-treatment data points of the control group were classified into the post-treatment intervention group.

The same procedure was applied for reaction time data, involving three-way interactions instead of two-way interactions. These three-way interactions included TREATMENT, TIME, and FLANKER or CUE condition for the Executive and Orienting Network performance, respectively.

## 3 Results

### 3.1 Descriptive statistics

Out of the 29 participants initially included in the study, 27 attended at least the first session and were included in the analyses. However, an additional 3 participants dropped out after the first and before the last session, resulting in 24 participants who completed the study entirely. Recruitment and testing were carried out between December 2022 and October 2023. Table 1 provides a description of the groups that underwent only the intervention and those who completed both the waitlist and intervention protocols.

**Table 1:** Sample description.

	Group waitlist (*n* = 13)		Group only intervention (*n* = 14)	
	Mean ± SD	Range	Mean ± SD	Range
Age	25.3 ± 7.0	18–37	22.6 ± 2.8	19–27
BMI in kg/m^2^	22.9 ± 4.5	18.3–32.2	22.9 ± 3.6	18.4–29.4
Gender	12f, 1m		14f	
Active menstrual cycle	11 yes, 2 no		14 yes	
HC usage	36.4%		42.9%	
PAF sum	54.7 ± 13.4	23–70	57.4 ± 20.2	19–93
BDI-II sum	12.9 ± 7.2	0–27	13.4 ± 8.0	2–30
Inclusion criterium	4 BDI, 4 PAF, 5 both		4 BDI, 4 PAF, 6 both	
DASS sum	34.1 ± 7,1	27–54	40.5 ± 8.3	25–50
RMSSD in ms	37.0 ± 10.9	19.6–51.5	41.9 ± 30.1	18.7–128.9
ANT-R executive in ms	148.1 ± 56.8	76.7–291.3	129.2 ± 39.1	80.8–194.9
ANT-R orienting in ms	85.0 ± 37.3	36.3–149.6	120.6 ± 35.1	46.7–183.8

The DASS, RMSSD, and ANT-R values were obtained during the first laboratory session of each participant. All other values were assessed during the screening. SD, standard deviation; BMI, body-mass-index; HC, hormonal contraceptives; PAF, premenstrual assessment form; BDI-II, Becks Depression Inventory; DASS, depression anxiety stress scales; RMSSD, root mean square of successive differences; ANT-R, revised attention network test.

There were 8 participants who were included due to elevated BDI measures, 8 due to elevated PAF20 measures, and 11 participants who fulfilled both inclusion criteria. Participants included due to PAF20 and BDI did not differ significantly in BDI values, *t*(14) = 1.32, *p* = .21, but did differ in PAF20 values, *t*(11) = 3.5, *p* < .01. Because the overall sample size was fairly small and because of the pilot nature of the study, we decided to include all participants in all analyses.

Three participants had to use the alternative HRVB system (“Qiu”) due to outdated OS versions on their phones. None of the results changed effect directions or significance levels when excluding these participants. A descriptive comparison of App and Qiu user outcomes as well as the main analysis results without the Qiu users can be found in the Supplementary S2.

### 3.2 Study compliance

Within the participants who completed the study, the mean practice frequency was 23.7 times, with a median of 24.5. The minimum practice frequency was 13 times, and the maximum was 51, with one practice session including 5 min of biofeedback.

### 3.3 Premenstrual symptoms

Out of 21 PAF20 post values recorded for the T5 measurement, 9 were replaced with the follow-up measurements. This occurred because there was either no premenstrual phase during the intervention period or the premenstrual phase occurred during the first two weeks of the intervention phase.

The final model for predicting premenstrual symptoms incorporated the TREATMENT and TIME variables along with their interaction. Furthermore, it included the SCALE of the PAF20 questionnaire to which each symptom belonged (psychological vs. physiological symptoms) and its interaction with the TREATMENT * TIME interaction (see Table 2). The final model was:

**Table 2:** Results of a linear mixed model predicting premenstrual symptoms.

Predictors	Value PAF20 item		
	Estimates	CI	*p*
(Intercept)	2.92	2.44 to 3.40	**<0**.**001**
Group [W]	−0.28	−0.50 to −0.07	**0**.**010**
Time	−0.30	−0.54 to −0.07	**0**.**012**
Scale [psy]	1.08	0.61 to 1.56	**<0**.**001**
Group [W] * time	0.40	0.03 to 0.78	**0**.**034**
[Group (I) * time] * scale [psy]	−0.36	−0.65 to −0.06	**0**.**017**
[Group (W) * time]* scale [psy]	0.13	−0.26 to 0.51	0.520
Random effects			
*σ* ^2^	1.40		
*τ*_00__participant_	0.58		
τ_00__item_	0.24		
ICC	0.37		
*N*_participant_	24		
*N*_item_	20		
Observations	1,300		
Marginal *R*^2^/Conditional *R*^2^	0.116/0.442		

*P*-values printed in bold indicate significant effects.

The random effect structure includes participant intercepts and item intercepts. PAF20 – premenstrual assessment form short version; group – treatment (biofeedback vs. waitlist); W – waitlist; I – intervention (biofeedback); psy – psychological symptoms.

Value ∼ treatment * time + scale + treatment:time:scale + (1|vpn) + (1|item).

**Figure 3:**
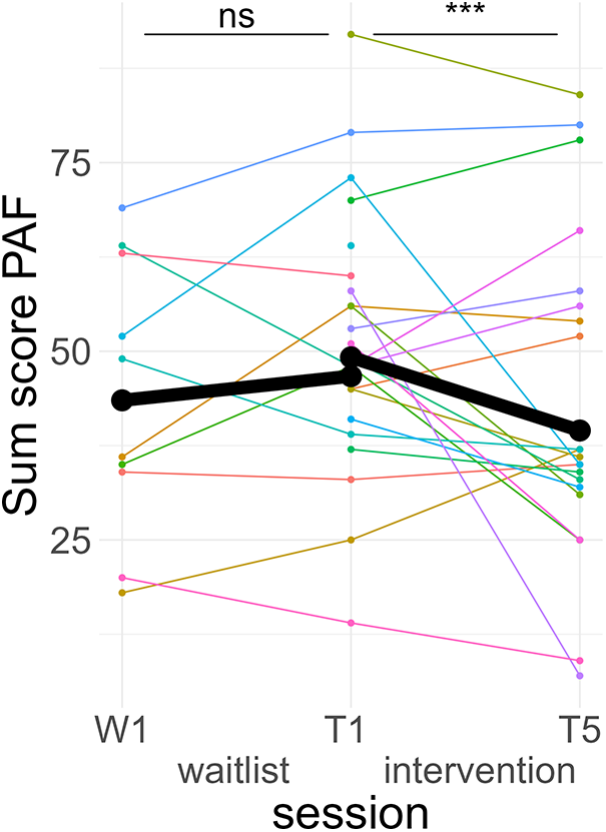
Course of premenstrual symptoms. Colored lines indicate individual participants. Black lines indicate the predicted interaction effect based on the mixed effect model depicted in Table 2. The waitlist period occurred between sessions W1 and T1, while the heart rate variability biofeedback intervention period took place between T1 and T5. PAF, premenstrual assessment form; ***, *p* < .001; ns, not significant.

*Post hoc* Tukey testing of the two-way interaction TREATMENT * TIME revealed a significant improvement in the intervention group, *d* = −0.30, *t*_ratio_ (1,258) = −5.89, *p* < .001, whereas there was no significant pre-post difference in the waitlist group, *d* = 0.10, *t*_ratio_ (1,252) = 1.35, *p* = .18 (see Figure 3). When SCALE was included in the interaction, it showed that the improvement in the intervention group was larger for psychological scale items (*d*_psych_ = −0.42) than for physiological scale items (*d*_physio_ = −0.19), with both improvements being significant. Detailed *post hoc* testing results for the TIME * TREATMENT * SCALE interaction can be found in Table 3.

**Table 3:** *Post hoc* tukey effects of time*treatment*scale interaction term predicting premenstrual symptoms.

	Scale	Diff	*p*
Waitlist	Physio	0.06	.54
	Psych	0.14	.11
Intervention	Physio	−0.19	.**012**
	Psych	−0.42	**<**.**0001**

*P*-values printed in bold indicate significant effects.

Diff – standardized pre-post treatment difference; psych – psychological symptoms; physio – physiological symptoms.

### 3.4 Depressive symptoms

The final model predicting depressive symptoms included only TREATMENT and TIME, as well as their interaction as fixed effects (see Table 4). The model was the following:

**Table 4:** Results of a linear mixed model predicting depressive symptoms.

Predictors	Value BDI item		
	Estimates	CI	*p*
(Intercept)	1.69	0.54 to 0.83	**<0**.**001**
Group [W]	−0.09	−0.18 to 0.00	0.054
Time	−0.17	−0.24 to −0.10	**<0**.**001**
Group [W] * time	0.21	0.09 to 0.33	**0**.**001**
Random effects			
*σ* ^2^	0.34		
τ_00__participant_	0.10		
τ_00__item_	0.03		
ICC	0.27		
*N*_participant_	27		
*N*_item_	21		
Observations	1,617		
Marginal *R*^2^/Conditional *R*^2^	0.010/0.281		

*P*-values printed in bold indicate significant effects.

The random effect structure includes participant intercepts and item intercepts. BDI – Beck's Depression Inventory II; group – treatment (biofeedback vs. waitlist); W – waitlist.

Value ∼ treatment*time + (1|vpn) + (1|item).

**Figure 4:**
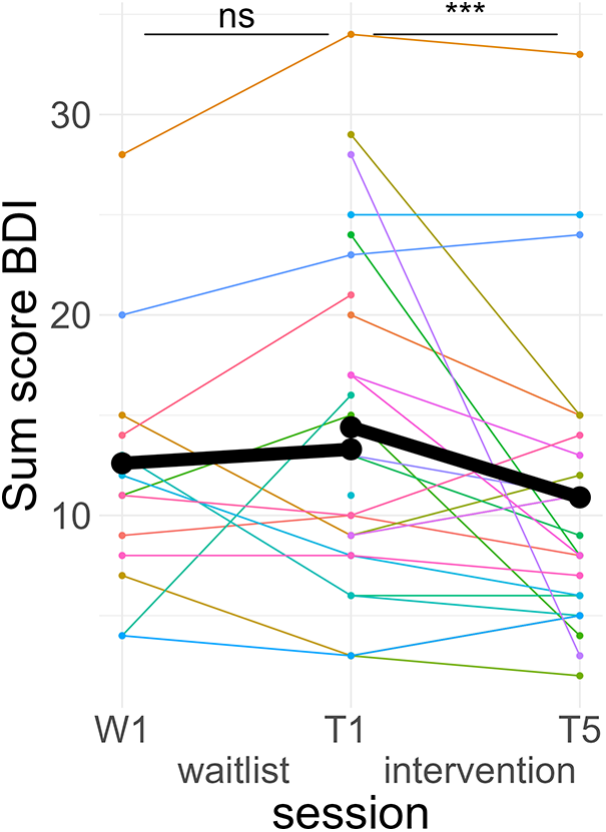
Course of depressive symptoms. Colored lines indicate individual participants. Black lines indicate the predicted interaction effect based on the mixed effect model depicted in Table 4. The waitlist period occurred between sessions W1 and T1, while the heart rate variability biofeedback intervention period took place between T1 and T5. BDI, Beck's Depression Inventory II; ***, *p* < .001; ns, not significant.

A *post hoc* Tukey test of the interaction showed that symptom scores significantly improved in the intervention period, *d* = −0.25, *t*_ratio_ (1,576) = −4.71, *p* < .001, but not in the wait-list period, *d* = 0.05, *t*_ratio_ (1,567) = 0.73, *p* = .46 (see Figure 4).

### 3.5 Stress and anxiety

The best model predicting DASS values included the RMSSD and the DASS SCALE (anxiety, stress or depression) on top of TREATMENT, TIME and their interaction (see Table 5). There was no three-way interaction of TREATMENT * TIME * SCALE. The model was:

**Table 5:** Results of a linear mixed model predicting stress/anxiety symptoms.

Predictors	Value DASS item		
	Estimates	CI	*p*
(Intercept)	1.12	0.64 to 1.60	**<0**.**001**
Time	−0.14	−0.22 to −0.07	**<0**.**001**
Group [W]	−0.11	−0.20 to −0.01	**0**.**023**
Scale [D]	0.11	−0.19 to 0.40	0.466
Scale [S]	0.44	0.15 to 0.74	**0**.**003**
rmssd	0.14	0.03 to 0.26	**0**.**015**
Time * group [W]	0.21	0.09 to 0.34	**0**.**001**
Random effects			
*σ* ^2^	0.35		
τ_00__participant_	0.14		
τ_00__item_	0.07		
ICC	0.39		
*N*_participant_	27		
*N*_item_	21		
Observations	1,617		
Marginal *R*^2^/Conditional *R*^2^	0.074/0.432		

*P*-values printed in bold indicate significant effects.

The random effect structure includes participant intercepts and item intercepts. DASS – Depression Anxiety and Stress Scale; group – treatment (biofeedback vs. waitlist); W – waitlist; D – depression scale; S – stress scale; RMSSD – root mean square of successive differences.

Value ∼ treatment*time + scale + RMSSD + (1|vpn) + (1|item).

**Figure 5:**
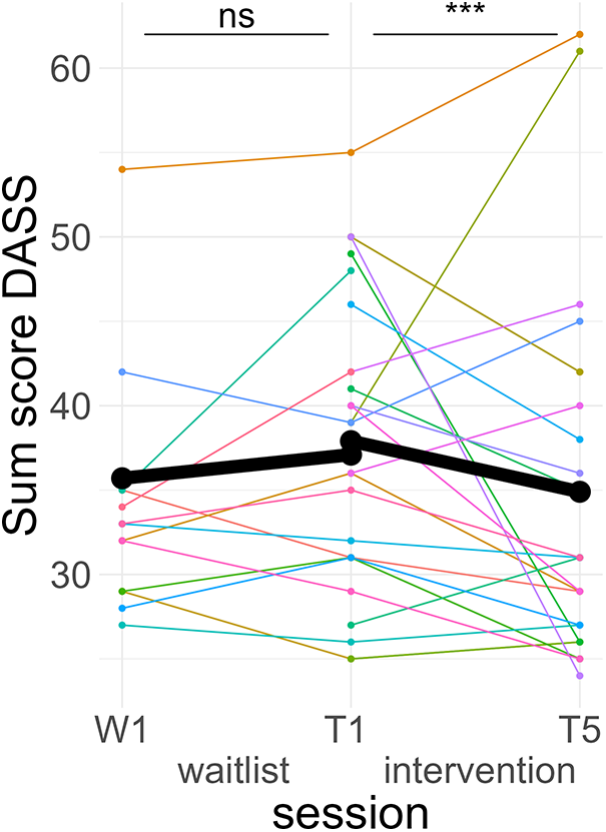
Course of anxiety/stress symptoms. Colored lines indicate individual participants. Black lines indicate the predicted interaction effect based on the mixed effect model depicted in Table 5. The waitlist period occurred between sessions W1 and T1, while the heart rate variability biofeedback intervention period took place between T1 and T5. DASS, Depression Anxiety and Stress Scales; ***, *p* < .001; ns, not significant.

*Post hoc* Tukey testing of the TREATMENT * TIME interactions showed a significant improvement in the intervention period, *d* = −0.19, *t*_ratio_ (1,573) = −3.88, *p* < .001, but not in the waitlist period, *d* = 0.09, *t*_ratio_ (1,567) = 1.37, *p* = .17 (see Figure 5).

### 3.6 Heart rate variability

The RMSSD was log-transformed to approximate a normal distribution, aligning it with the methodology used in other vmHRV research studies. Two participants had to be excluded from the post measurement, as they had acute respiratory tract infections.

The final model predicting the log(RMSSD) included only TREATMENT, TIME and the TREATMENT * TIME interaction as fixed effects (see Table 6).

**Table 6:** Results of a linear mixed model predicting vagally mediated heart rate variability.

Predictors	log(RMSSD in ms)		
	Estimates	CI	*p*
(Intercept)	3.53	3.32 to 3.75	**<0**.**001**
group [W]	0.07	−0.15 to 0.29	0.546
time	0.06	−0.12 to 0.23	0.509
group [W] * time	−0.13	−0.42 to 0.16	0.381
Random effects			
*σ* ^2^	0.08		
τ_00__participant_	0.21		
ICC	0.71		
*N*_participant_	25		
Observations	71		
Marginal *R*^2^/Conditional *R*^2^	0.003/0.711		

*P*-values printed in bold indicate significant effects.

The random effect structure includes participant intercepts. RMSSD – root mean square of successive differences; group – treatment (biofeedback vs. waitlist); W – waitlist.

Value ∼ treatment*time + (1|vpn) + (1|item).

**Figure 6:**
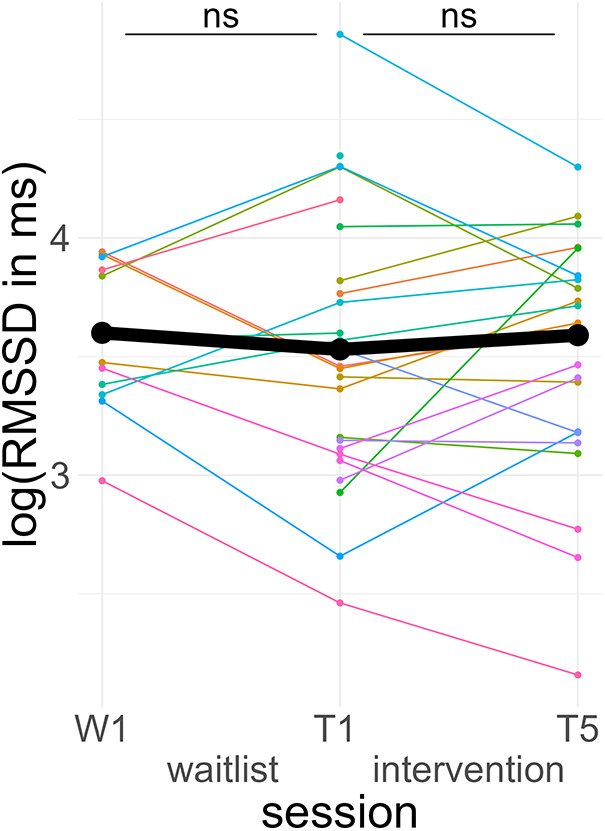
Course of vagally mediated heart rate variability. Colored lines indicate individual participants. Black lines indicate the predicted interaction effect based on the mixed effect model depicted in Table 6. The waitlist period occurred between sessions W1 and T1, while the heart rate variability biofeedback intervention period took place between T1 and T5. RMSSD, root mean square of successive differences; ns, not significant.

None of the fixed effects were significant predictors. A *post hoc* Tukey test of the interaction confirmed no significant improvement in the intervention group, *d* = 0.11, *t*_ratio_ (44) = 0.66, *p* = .51, and no significant pre-post difference in the waitlist group, *d* = −0.14, *t*_ratio_ (43) = 0.61, *p* = .55 (see Figure 6).

### 3.7 Attentional control

The reaction time prediction model included main effects for TIME, TREATMENT, CUE, and FLANKER. Additionally, two three-way interactions were included—TREATMENT * TIME * CUE and TREATMENT * TIME * FLANKER. Both of these three-way interactions revealed significant terms in the model (see Table 7).

**Table 7:** Results of a linear mixed model predicting attentional control.

Predictors	Reaction time		
	Estimates	CI	*p*
(Intercept)	600.31	574.20 to 626.41	**<0**.**001**
Time	−37.63	−49.22 to −26.05	**<0**.**001**
Group [W]	39.00	31.02 to 46.99	**<0**.**001**
Flanker [incongruent]	130.14	123.20 to 137.08	**<0**.**001**
Cue [valid]	−98.44	−106.51 to −90.37	**<0**.**001**
Time × group [I] × cue [invalid]	45.56	28.15 to 62.97	**<0**.**001**
Time × group [W] ×cue [invalid]	−0.22	−16.33 to 15.89	0.978
Time × group [I] × cue [valid]	41.09	27.57 to 54.61	**<0**.**001**
Time × group [I] × flanker [incongruent]	−26.57	−37.90 to −15.25	**<0**.**001**
Time × group [W] × flanker [incongruent]	−9.44	−23.31 to 4.43	0.182
Random effects			
*σ* ^2^	11,369.21		
τ_00__participant_	4,325.41		
ICC	0.28		
*N*_participant_	27		
Observations	7,040		
Marginal *R*^2^/Conditional *R*^2^	0.267/0.469		

*P*-values printed in bold indicate significant effects.

The model predicts trial-based prediction times of the revised attention network test. The random effect structure includes participant intercepts. Group – treatment (biofeedback vs. waitlist); W – waitlist; I – intervention (biofeedback).

Reaction Time ∼ treatment:time:cue + treatment:time:flanker + time + treatment + cue + flanker + (1|vpn) + (1|item).

We used linear trend estimates from the emmeans package (v1.8.8) to compare valid vs. invalid (cue) and congruent vs. incongruent (flanker) slopes in each pre-post comparison. This approximated the original score calculations. Significant differences in slopes indicate changes in the Orienting Score (invalid vs. valid cue trials) or Executive Score (incongruent vs. congruent flanker trials) between pre and post-assessment.

**Figure 7:**
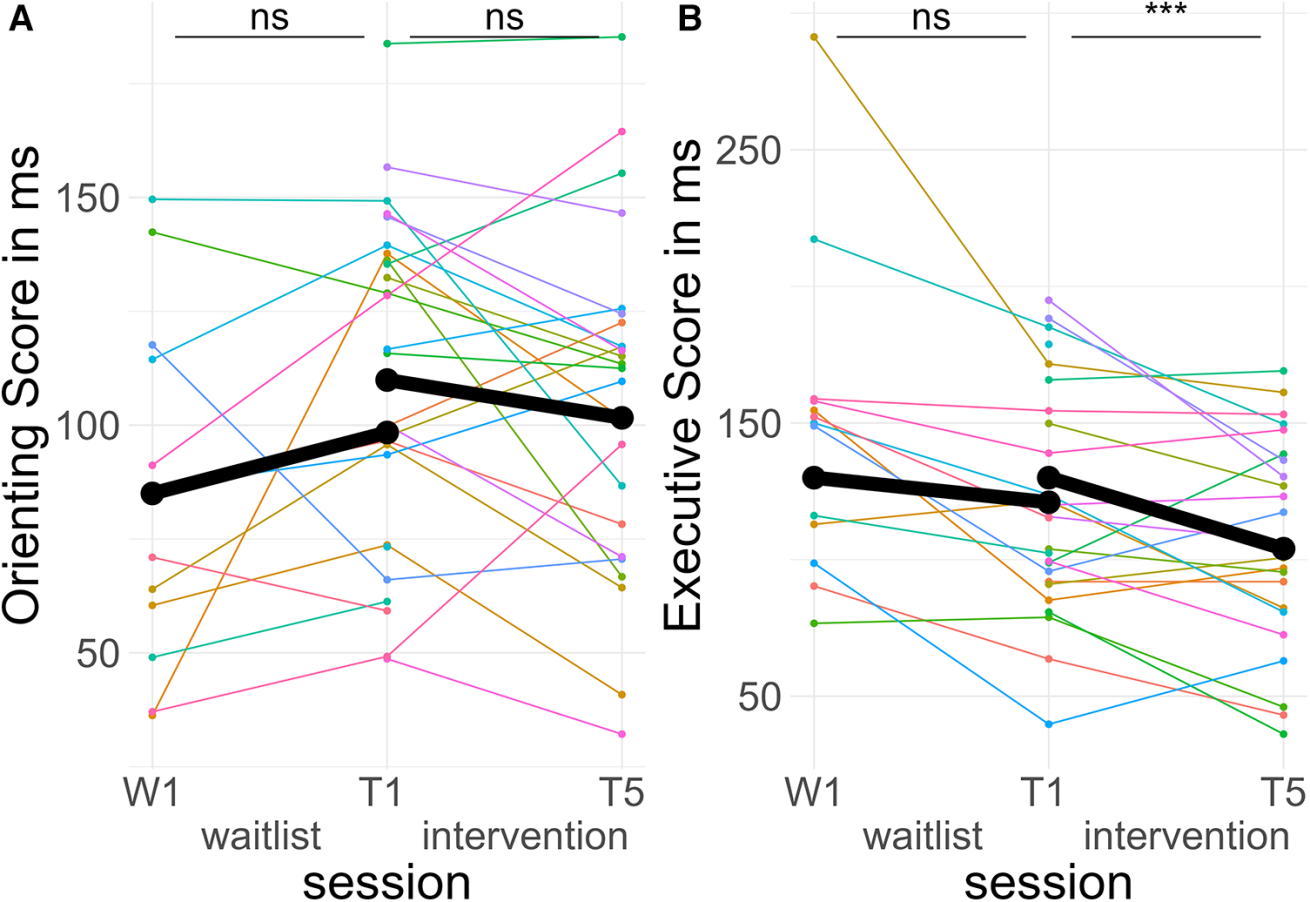
Course of attentional control. Colored lines indicate individual participants. Black lines indicate the predicted interaction effect based on the mixed effect model depicted in Table 5. The waitlist period occurred between sessions W1 and T1, while the heart rate variability biofeedback intervention period took place between T1 and T5. (**A**) Orienting Score—(reaction time _incongruent spatial cues_—reaction time _congruent spatial cues_); (**B**) Executive Score—(reaction time _invalid flankers_—reaction time _valid flankers_). ***, *p* < .001; ns, not significant.

In the TIME * TREATMENT * CUE interaction, there were no significant differences in pre-post slopes between valid and invalid trials during both the intervention period, *d* = −0.03, *z*_ratio_ = −0.67, *p* = .91, and the waitlist period, *d* = 0.00, *z*_ratio_ = 0.03, *p* = 1, (see Figure 7A).

In the TIME * TREATMENT * FLANKER interaction, there was a significant difference in pre-post slopes between congruent and incongruent trials during the intervention period, *d* = −0.18, *z*_ratio_ = −4.60, *p* < .001, but no difference in the waitlist period, *d* = −0.06, *z*_ratio_ = −1.34, *p* = .54. The effect in the intervention period indicated less difference between valid and invalid trials post-treatment compared to pre-treatment, equivalent to better Executive Scores (see Figure 7B).

## 4 Discussion

In this study, we investigated the impact of a 4-week photoplethysmography smartphone-based HRVB intervention on premenstrual and depressive symptoms using a waitlist-control design. Additionally, we assessed its effects on stress symptoms, vmHRV, and attentional control. Our findings revealed improvements in premenstrual, depressive, and stress symptoms following the intervention, with no significant changes observed during the waitlist period. These results confirmed our first three hypotheses (H1-H3). However, we did not observe any effects on vmHRV, and the results for attentional control were mixed. We therefore had to reject hypothesis H4 (no improvements in vmHRV) and H5a (no improvements in Orienting attentional control) in this current sample, while accepting H5b (immprovements in Executive Functioning attentional control).

The positive effects on premenstrual, depressive, anxiety, and stress symptoms found in our study highlight the feasibility and effectiveness of a smartphone-based HRVB intervention without the need for external devices. Laborde et al. (64) reported similar effects of a slow-paced breathing intervention, whether or not visual feedback on current HRV was provided. This suggests that slow-paced breathing is the primary driver of the intervention's effectiveness. However, learning the technique of abdominal slow-paced breathing, which allows for a relaxed resonance frequency activation, can be challenging. The drastically slowed-down breathing can be uncomfortable and may even lead to hyperventilation (65). Providing visual feedback on respiratory sinus arrhythmia can assist users in correctly employing the method at home without extensive training. Moreover, this biofeedback feature can enhance user engagement with the app, as it offers immediate visual results of the breathing intervention, providing a sense of immediate gratification. Essentially, this form of feedback incorporates a simplified version of gamification into the intervention, which can increase motivation and engagement (66).

Our primary objective was to investigate whether HRVB could help alleviate PMS symptoms. We observed a significant improvement in the premenstrual phase following 2–4 weeks of HRVB practice compared to the preceding cycle in which no HRVB was practiced. The effect size we observed is approximately 0.3, with a slightly larger effect of 0.4 in the psychological symptom scale. In this context, the effect size is similar to the moderate effect size found in meta-analyses of depressive symptoms (3). This finding supports the notion that PMS may result from an altered reactivity within the ALLO/GABA system (27), affecting the CAN as proposed by Thayer and Lane (11). Consequently, interventions designed to target the interconnectivity and functional capacity of the CAN may hold promise in improving PMS symptoms.

However, further studies with expanded paradigms are needed to determine whether this effect is mediated by a reduction in stress throughout the menstrual cycle or by an increase in the capacity of the CAN, which may buffer modulatory fluctuations throughout the cycle. It also remains unclear whether there is a critical phase in the menstrual cycle when HRVB has a more significant impact on subsequent premenstrual symptoms. It is possible that practicing HRVB throughout the preceding follicular phase reduces chronic stress (2) and, consequently, chronic ALLO exposure. This prolonged exposure has been suggested to contribute to atypical GABA receptor reactivity to ALLO fluctuations later in the cycle, which is assumed to cause PMS (27). Another possibility is that during the premenstrual phase, the acute stress-relieving effects [see e.g., (67, 68)] of HRVB directly alleviate the symptom burden.

Independently of mechanisms of action, HRVB is an easily learned intervention with minimal to no side effects, offering PMS sufferers an opportunity to enhance their symptom management self-efficacy. Furthermore, it can lower the threshold for receiving treatment. When delivered through a smartphone application, it becomes even more accessible and, if its effectiveness is established, can be seamlessly integrated into widely used menstrual cycle-related health apps.

Our results also demonstrated improvements in depressive symptoms, as well as anxiety and stress symptoms, through the smartphone-based HRVB intervention. This aligns with prior research using other HRVB intervention methods (2, 3), although the effects we observed are somewhat smaller than the meta-analytic effects on depression and anxiety/stress. This discrepancy may be due to ceiling effects. The majority of studies investigating HRVB effects typically focus on clinical populations with very high values in the respective outcome measures. Our inclusion criteria, on the other hand, involved individuals with above-average PMS symptoms or slightly elevated depressive symptoms. 30% of the participants were included in our study due to only PMS symptoms. While clinically significant affective premenstrual symptoms (premenstrual dysphoric disorder) are often comorbid with anxiety and depression (69), our subclinical sample likely had lower baseline scores in both BDI-II and DASS values compared to the samples in most studies included in the meta-analysis. This could account for the slightly smaller effect sizes observed in our study.

We did not observe improvements in vmHRV (RMSSD) following the HRVB intervention. Contrary to our findings, Laborde et al. (10) reported consistent elevations in vmHRV parameters after HRVB and slow-paced breathing interventions in their meta-analysis. The effect size for this improvement was approximately *Hedges' g* = 0.3. Notably, this effect size is smaller than the meta-analytic effect sizes for depressive symptoms [*g* = 0.4; (3)] or anxiety/stress [*g* = 0.8; (2)]. These findings suggest that improvements in vmHRV alone do not fully account for the observed affective and cognitive effects. This is consistent with other findings that demonstrate physiological and clinical outcomes do not always change simultaneously in HRVB (70). Critically, our study may not have detected this effect due to its relatively small sample size. A G*Power analysis indicates that a sample size of more than 70 would be required to replicate the effect of 0.3 with a power of.8. Our sample size was significantly below this threshold. The reason why effects were found in other outcome measures lies in the advantage of mixed model analysis, which allows us to include each item/trial individually without the need to condense the information into a composite score. This significantly increases the number of data points included, by 20 to 144 times, depending on the measurement. However, in the case of vmHRV, it is necessary compute a single value per measurement, which drastically reduces statistical power. This suggests the possibility that HRVB may have had a beneficial impact on vmHRV, but our study may have been underpowered to detect this effect.

The influence of the HRVB on the attentional control domains yielded mixed results. We chose to employ the Executive and Orienting Scores of the ANT-R as outcome measures, given their documented correlations with vmHRV (23, 71–73). Interestingly, despite previous studies (71, 73) indicating a stronger association between the Orienting Score and vmHRV (compared to the Executive Score vmHRV association), we did not observe any improvements in this measure. However, we did find enhancements in the Executive Score following the intervention period. These varying outcomes align with the findings of a meta-analysis conducted by Tinello et al. (22), who reported positive effects of HRVB on Executive Functions in approximately half of the studies they reviewed, with a slightly higher likelihood of effects in the attention domain. This highlights the need for further research to elucidate the specific impact of HRVB on cognitive outcomes.

Although we found significant beneficial effects of smartphone-based HRVB on several mental health and some cognitive outcomes, our study has several limitations that need to be considered. Firstly, the most notable limitation is our small sample size. Although linear mixed model analyses help to overcome the limited statistical power due to small samples by including individual items/trials in the analysis, it is important to interpret the results and their applicability to larger populations with caution. This is a major weakness of the study, and therefore it is crucial that the results are replicated in a larger sample to confirm the effects. Additionally, a part of the sample (3 participants) had to use alternative HRVB systems due to outdated OS versions. Although the significance and direction of the effects did not change when excluding those data points (see Supplementary S2), this further reduces the interpretability of the effects concerning PPG and smartphone-based HRVB effectiveness. Another limitation is related to the passive control group, which has methodological weaknesses. In future studies, it would be beneficial to implement active control groups to better distinguish the intervention's effects from any potential placebo effects. Additionally, we did not conduct clinical assessments of PMS or depression. However, it is essential to note that our study was not aimed at identifying treatments for a clinically relevant premenstrual dysphoric disorder. Instead, our primary focus was on investigating a user-friendly intervention to help individuals self-manage premenstrual symptoms, regardless of their severity levels. Furthermore, our study included participants regardless of their use of hormonal contraceptives and their current menstrual cycle phase. For future studies, it is advisable to standardize these criteria to ensure a more consistent assessment.

## 5 Conclusion

In summary, smartphone-based HRVB has proven to be effective in enhancing both emotional and cognitive well-being, without the need for external devices. This intervention holds promise as a novel approach for self-managing premenstrual symptoms and provides a more accessible solution for harnessing the known benefits of HRVB for depressive, stress, and anxiety symptoms, as well as certain aspects of attention.

## Data Availability

The raw data supporting the conclusions of this article will be made available by the authors, without undue reservation.
