# Supplementary material for: Alleviating premenstrual symptoms with smartphone-based heart rate variability biofeedback training: a pilot study
Source: Front Digit Health. 2024 Jun 14;6:1337667. doi: 10.3389/fdgth.2024.1337667 (PMC11211633; doi:10.3389/fdgth.2024.1337667)
Supplement: Supplementary file 1 [file Table1.docx]

**Supplement S1 *Smartphones used by participants***

**Smartphone n**

Apple iPhone11 5

Apple iPhone12Mini 2

Apple iPhone13 1

Apple iPhone13Pro 1

Apple iPhone7 1

Apple iPhone8 2

Apple iPhone8Plus 1

Apple iPhoneSE2 1

Apple iPhoneXS 2

Apple unknown 3

HUAWEI EML-L29 1

samsung SM-A525F 1

samsung SM-A715F 1

samsung SM-N770F 1
