# Supplementary material for: Alleviating premenstrual symptoms with smartphone-based heart rate variability biofeedback training: a pilot study
Source: Front Digit Health. 2024 Jun 14;6:1337667. doi: 10.3389/fdgth.2024.1337667 (PMC11211633; doi:10.3389/fdgth.2024.1337667)
Supplement: Supplementary file 2 [file Table2.docx]

**Supplement S2 *Statistics excluding Qiu users***

Table S2.1. Results of a linear mixed model predicting premenstrual symptoms without Qiu users

|  | **value PAF20 item** | | |
| --- | --- | --- | --- |
| *Predictors* | *Estimates* | *CI* | *p* |
| (Intercept) | 3.02 | 2.52 – 3.51 | **<0.001** |
| group [W] | -0.26 | -0.51 – -0.01 | **0.038** |
| time | -0.34 | -0.61 – -0.08 | **0.010** |
| scale [psy] | 1.07 | 0.59 – 1.56 | **<0.001** |
| group [W] * time | 0.35 | -0.07 – 0.77 | 0.101 |
| (group [I] * time) * scale [psy] | -0.42 | -0.74 – -0.09 | **0.012** |
| (group [W] * time )* scale [psy] | 0.20 | -0.23 – 0.64 | 0.359 |
| **Random Effects** | | | |
| σ^2^ | 1.47 | | |
| τ_00_ _vpn_ | 0.52 | | |
| τ_00_ _item_ | 0.24 | | |
| ICC | 0.34 | | |
| N _vpn_ | 21 | | |
| N _item_ | 20 | | |
| Observations | 1100 | | |
| Marginal R^2^ / Conditional R^2^ | 0.119 / 0.421 | | |

Note. The random effect structure includes participant intercepts and item intercepts. PAF20 – premenstrual assessment form short version; group – treatment (biofeedback vs. waitlist); W – waitlist; I – intervention (biofeedback); psy – psychological symptoms.

Table S2.2. Results of a linear mixed model predicting depressive symptoms without Qiu users

|  | **value BDI item** | | |
| --- | --- | --- | --- |
| *Predictors* | *Estimates* | *CI* | *p* |
| (Intercept) | 1.69 | 1.53 – 1.84 | **<0.001** |
| group [W] | -0.11 | -0.20 – -0.01 | **0.033** |
| time | -0.17 | -0.25 – -0.10 | **<0.001** |
| group [W] * time | 0.23 | 0.10 – 0.35 | **0.001** |
| **Random Effects** | | | |
| σ^2^ | 0.33 | | |
| τ_00_ _participant_ | 0.11 | | |
| τ_00_ _item_ | 0.03 | | |
| ICC | 0.28 | | |
| N _participant_ | 24 | | |
| N _item_ | 21 | | |
| Observations | 1407 | | |
| Marginal R^2^ / Conditional R^2^ | 0.011 / 0.292 | | |

Note. The random effect structure includes participant intercepts and item intercepts. BDI – Beck’s Depression Inventory II; group – treatment (biofeedback vs. waitlist); W – waitlist.

Table S2.3. Results of a linear mixed model predicting vagally mediated heart rate variability without Qiu users

|  | **log(RMSSD in ms)** | | |
| --- | --- | --- | --- |
| *Predictors* | *Estimates* | *CI* | *p* |
| (Intercept) | 3.55 | 3.31 – 3.79 | **<0.001** |
| group [W] | 0.04 | -0.22 – 0.29 | 0.775 |
| time | 0.05 | -0.14 – 0.25 | 0.576 |
| group [W] * time | -0.09 | -0.43 – 0.24 | 0.577 |
| **Random Effects** | | | |
| σ^2^ | 0.09 | | |
| τ_00_ _participant_ | 0.23 | | |
| ICC | 0.71 | | |
| N _participant_ | 22 | | |
| Observations | 61 | | |
| Marginal R^2^ / Conditional R^2^ | 0.002 / 0.709 | | |

Note. The random effect structure includes participant intercepts. RMSSD – root mean square of successive differences; group – treatment (biofeedback vs. waitlist); W – waitlist.

Table S2.4. Descriptive differences of outcome variables between App users and Qiu users.

|  |  | PAF20 | BDI | DASS | log(RMSSD) | Exe in ms | Orient in ms |
| --- | --- | --- | --- | --- | --- | --- | --- |
| App users mean ±sd | Pre | 53.2±15.9 | 14.7±8.9 | 38.0±8.7 | 3.5±0.6 | 122.2±42.6 | 111.1±37.4 |
|  | Post | 43.8±21.5 | 10.8±7.9 | 35.1±11.0 | 3.5±0.5 | 107.3±39.3 | 105.0±34.7 |
| Qiu user 1 | Pre | 45 | 20 | 50 | 3.8 | 92.0 | 99.8 |
|  | Post | 52 | 15 | 42 | 4.0 | 92.0 | 122.5 |
| Qiu user 2 | Pre | 56 | 9 | 36 | 3.4 | 121.5 | 73.7 |
|  | Post | 54 | 11 | 29 | 3.7 | 82.3 | 40.8 |
| Qiu user 3 | Pre | 14 | 8 | 29 | 3.1 | 139.0 | 128.5 |
|  | post | 9 | 7 | 25 | 2.8 | 147.5 | 164.5 |

Note. Pre (T1) and post (T5) values of App users (n=24) and Qiu users (n=3). SD – standard deviation; PAF20 – premenstrual assessment form short version; BDI – Beck’s Depression Inventory II; DASS – depression anxiety stress scales; RMSSD – root mean square of successive differences; Exe – Executive Functioning Network Score of the revised Attention Network Test; Orient – Orienting Network Score of the revised Attention Network Test.
